# Supplementary material for: Molecular action of pyriproxyfen: Role of the Methoprene-tolerant protein in the pyriproxyfen-induced sterilization of adult female mosquitoes
Source: PLoS Negl Trop Dis. 2020 Aug 31;14(8):e0008669. doi: 10.1371/journal.pntd.0008669 (PMC7485974; doi:10.1371/journal.pntd.0008669)
Supplement: S1 Fig — After adult emergence, both male and female Ae. aegypti mosquitoes were allowed to mate for three days. Female mosquitoes were treated with PPF (70 μg/cm2) for 30 minutes at the indicated time points. All the mosquitoes were given a blood meal at 120 h after eclosion. Follicle examination was performed at 48 h PBM. Oviposited eggs were counted six days after blood feeding. Matured eggs were allowed to hatch for seven days to record the hatching rate. PE, Post eclosion; PBM, Post blood-meal. (PDF) [file pntd.0008669.s001.pdf]

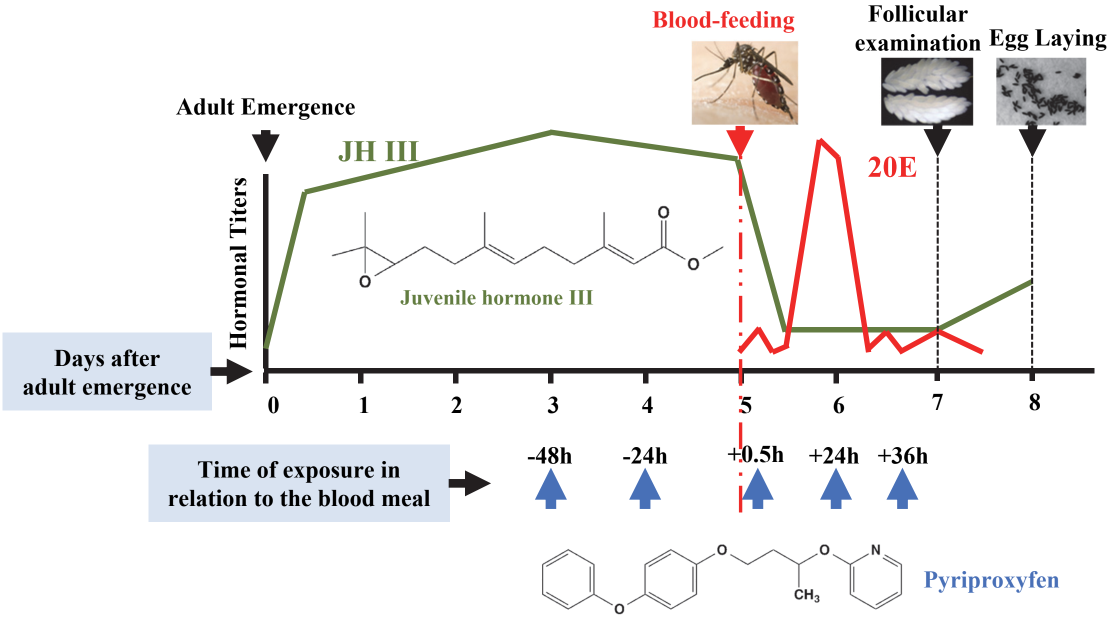

S1 Fig. Schematic diagram of PPF exposure at various stages in adult female mosquitoes. After adult emergence, both male and female *Ae. aegypti* mosquitoes were allowed to mate for three days. Female mosquitoes were treated with PPF (70 µg/cm^2) for 30 minutes at the indicated time points. All the mosquitoes were given a blood meal at 120 h after eclosion. Follicle examination was performed at 48 h PBM. Oviposited eggs were counted six days after blood feeding. Matured eggs were allowed to hatch for seven days to record the hatching rate. PE, Post eclosion; PBM, Post blood-meal.
