# Supplementary material for: Molecular action of pyriproxyfen: Role of the Methoprene-tolerant protein in the pyriproxyfen-induced sterilization of adult female mosquitoes
Source: PLoS Negl Trop Dis. 2020 Aug 31;14(8):e0008669. doi: 10.1371/journal.pntd.0008669 (PMC7485974; doi:10.1371/journal.pntd.0008669)
Supplement: S2 Fig — Adult female mosquitoes were exposed to PPF at the indicated time points. Mosquitoes from untreated, cyclohexane-treated, and PFF-treated groups were dissected six days after blood-feeding. Developed follicles inside the abdomen after oviposition were counted as ‘retained eggs’. Reported here are the average of retained eggs in each female mosquito (10 mosquitoes/ replicate) and the percentages of females with retained eggs (10 mosquitoes/ replicate). Results shown are the mean ± S.D. of three replicates. Statistical differences between the PPF-treated and cyclohexane-treated mosquitoes were analyzed using paired t-test (ns, p > 0.05; *, p < 0.05; **, p < 0.01; ***, p < 0.001). (PDF) [file pntd.0008669.s002.pdf]

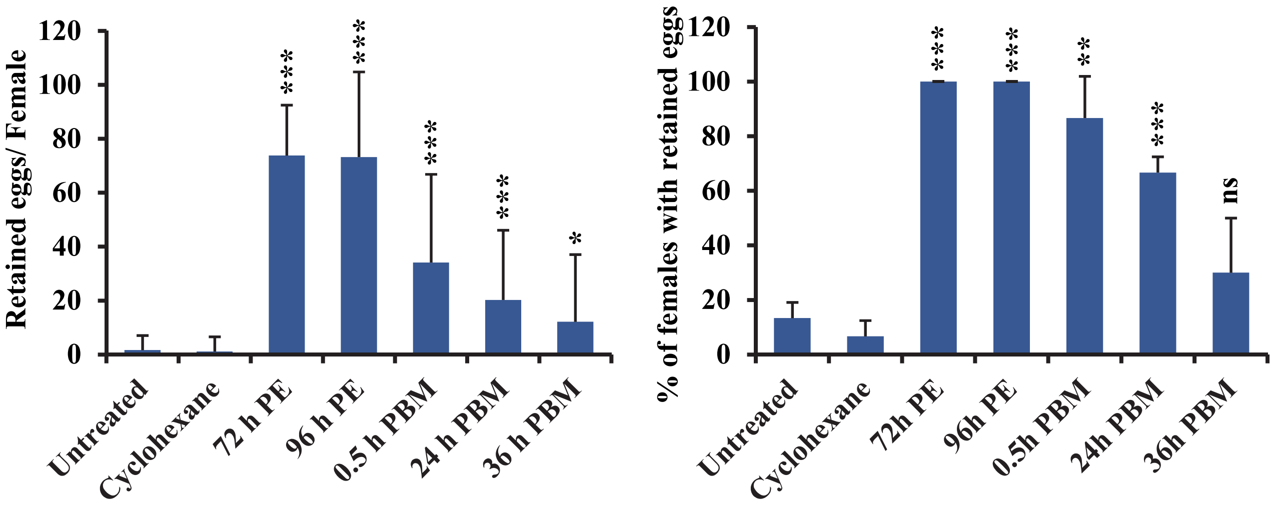

S2 Fig. Egg retention after different PPF treatments. Adult female mosquitoes were exposed to PPF at the indicated time points. Mosquitoes from untreated, cyclohexane-treated, and PFF-treated groups were dissected six days after blood-feeding. Developed follicles inside the abdomen after oviposition were counted as 'retained eggs'. Reported here are the average of retained eggs in each female mosquito (10 mosquitoes/ replicate) and the percentages of females with retained eggs (10 mosquitoes/ replicate). Results shown are the mean ± S.D. of three replicates. Statistical differences between the PPF-treated and cyclohexane-treated mosquitoes were analyzed using paired t-test (ns, $p > 0.05$; *, $p < 0.05$; **, $p < 0.01$; ***, $p < 0.001$).
