# Supplementary material for: Molecular action of pyriproxyfen: Role of the Methoprene-tolerant protein in the pyriproxyfen-induced sterilization of adult female mosquitoes
Source: PLoS Negl Trop Dis. 2020 Aug 31;14(8):e0008669. doi: 10.1371/journal.pntd.0008669 (PMC7485974; doi:10.1371/journal.pntd.0008669)
Supplement: S3 Fig — Female adults were exposed to PPF (70 μg/cm2) at 72 h PE and given a blood meal at 120 h PE. Thirty mosquitoes, from untreated, cyclohexane-treated, and PPF-treated groups, were dissected at 48 h PBM to examine ovarian follicles. Eggs were collected at 144 h PBM and imaging was performed using Leica Application Suite (v4.5). Scale bars represent 0.1 mm in the upper and lower panels. PBM, Post blood-meal. (PDF) [file pntd.0008669.s003.pdf]

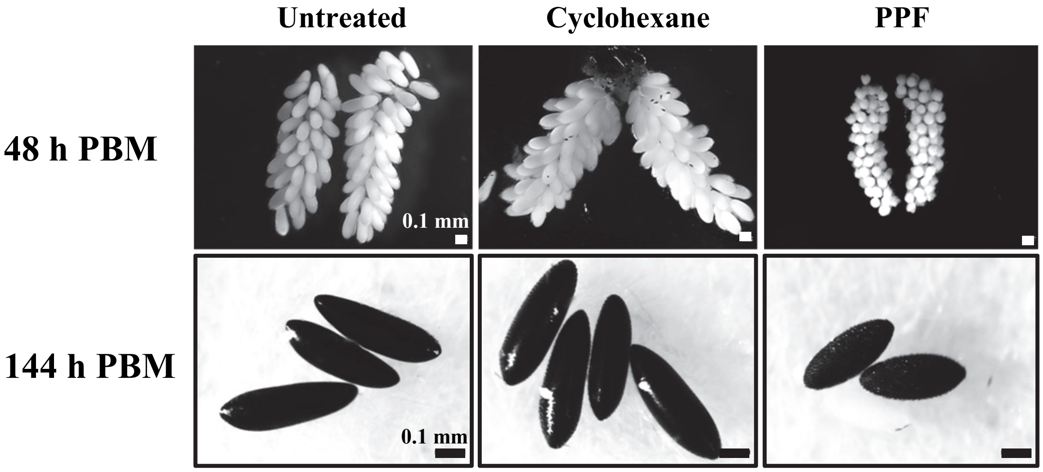

S3 Fig. Effect of PPF exposure on the morphology of follicles and eggs. Female adults were exposed to PPF (70 μg/cm^2) at 72 h PE and given a blood meal at 120 h PE. Thirty mosquitoes, from untreated, cyclohexane-treated, and PPF-treated groups, were dissected at 48 h PBM to examine ovarian follicles. Eggs were collected at 144 h PBM and imaging was performed using Leica Application Suite (v4.5). Scale bars represent 0.1 mm in the upper and lower panels. PBM, Post blood-meal.
