# Supplementary material for: Molecular action of pyriproxyfen: Role of the Methoprene-tolerant protein in the pyriproxyfen-induced sterilization of adult female mosquitoes
Source: PLoS Negl Trop Dis. 2020 Aug 31;14(8):e0008669. doi: 10.1371/journal.pntd.0008669 (PMC7485974; doi:10.1371/journal.pntd.0008669)
Supplement: S4 Fig — Adult female mosquitoes were treated with PPF at 72 h PE. The lengths of primary follicles were measured at the indicated time points throughout the first gonotrophic cycle. Error bars represent the standard deviation (SD) of three biological replicates. Statistical analysis was performed by paired t-test. Asterisks indicate significant differences in follicular length between cyclohexane-treated and PPF-treated groups (ns, p > 0.05; *, p < 0.05; **, p < 0.01; ***, p < 0.001). PE, Post-eclosion; PBM, Post blood-meal. (PDF) [file pntd.0008669.s004.pdf]

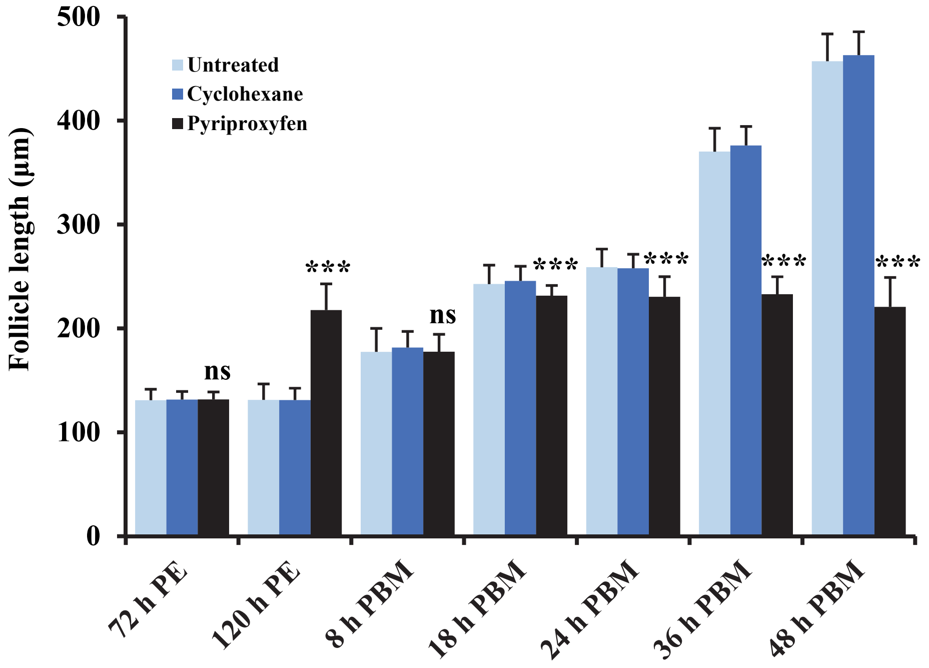

S4 Fig. Growth of primary follicles after PPF exposure. Adult female mosquitoes were treated with PPF at 72 h PE. The lengths of primary follicles were measured at the indicated time points throughout the first gonotrophic cycle. Error bars represent the standard deviation (SD) of three biological replicates. Statistical analysis was performed by paired t-test. Asterisks indicate significant differences in follicular length between cyclohexane-treated and PPF-treated groups (ns, $p > 0.05$; *, $p < 0.05$; **, $p < 0.01$; ***, $p < 0.001$). PE, Post-eclosion; PBM, Post blood-meal.
