# Supplementary material for: Molecular action of pyriproxyfen: Role of the Methoprene-tolerant protein in the pyriproxyfen-induced sterilization of adult female mosquitoes
Source: PLoS Negl Trop Dis. 2020 Aug 31;14(8):e0008669. doi: 10.1371/journal.pntd.0008669 (PMC7485974; doi:10.1371/journal.pntd.0008669)
Supplement: S5 Fig — PPF treatment was carried out at 72 h PE in Ae. aegypti female mosquitoes. Shown here with an asterisk sign (*) is a representative image of aborted ovarian follicles in PPF-treated mosquitoes at 48 h PBM. These follicles are characterized by their round shape, advance development of secondary follicle/prominent germarium, and the presence of intact nurse cells, which normally disappear in control mosquitoes at this stage. 1°, primary follicle; 2°, secondary follicle; G, germarium; NC, nurse cell. (PDF) [file pntd.0008669.s005.pdf]

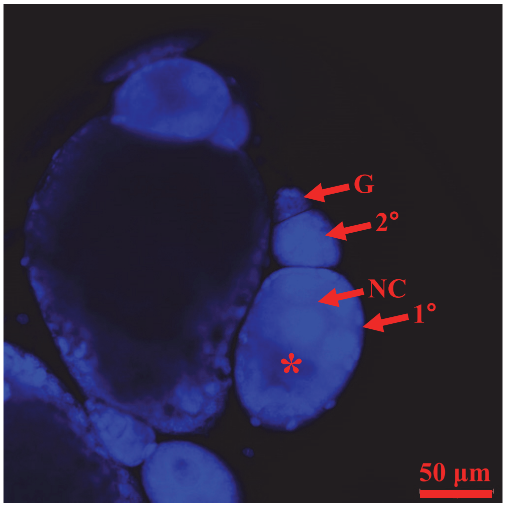

S5 Fig. PPF hampered follicular development of mosquitoes. PPF treatment was carried out at 72 h PE in *Ae. aegypti* female mosquitoes. Shown here with an asterisk sign (*) is a representative image of aborted ovarian follicles in PPF-treated mosquitoes at 48 h PBM. These follicles are characterized by their round shape, advance development of secondary follicle/prominent germarium, and the presence of intact nurse cells, which normally disappear in control mosquitoes at this stage. 1°, primary follicle; 2°, secondary follicle; G, germarium; NC, nurse cell.
