# Supplementary material for: Molecular action of pyriproxyfen: Role of the Methoprene-tolerant protein in the pyriproxyfen-induced sterilization of adult female mosquitoes
Source: PLoS Negl Trop Dis. 2020 Aug 31;14(8):e0008669. doi: 10.1371/journal.pntd.0008669 (PMC7485974; doi:10.1371/journal.pntd.0008669)
Supplement: S6 Fig — Adult female mosquitoes were treated with PPF (70 μg/cm2) at 72 h PE. Cyclohexane was used as a solvent control. RNA-seq analyses were performed using mosquito fat body and ovary tissues collected at 120 h PE and 24 h PBM. The percentage of differentially expressed genes in discrete functional categories is displayed in the pie chart. PE, Post eclosion; PBM, Post blood-meal. (PDF) [file pntd.0008669.s006.pdf]

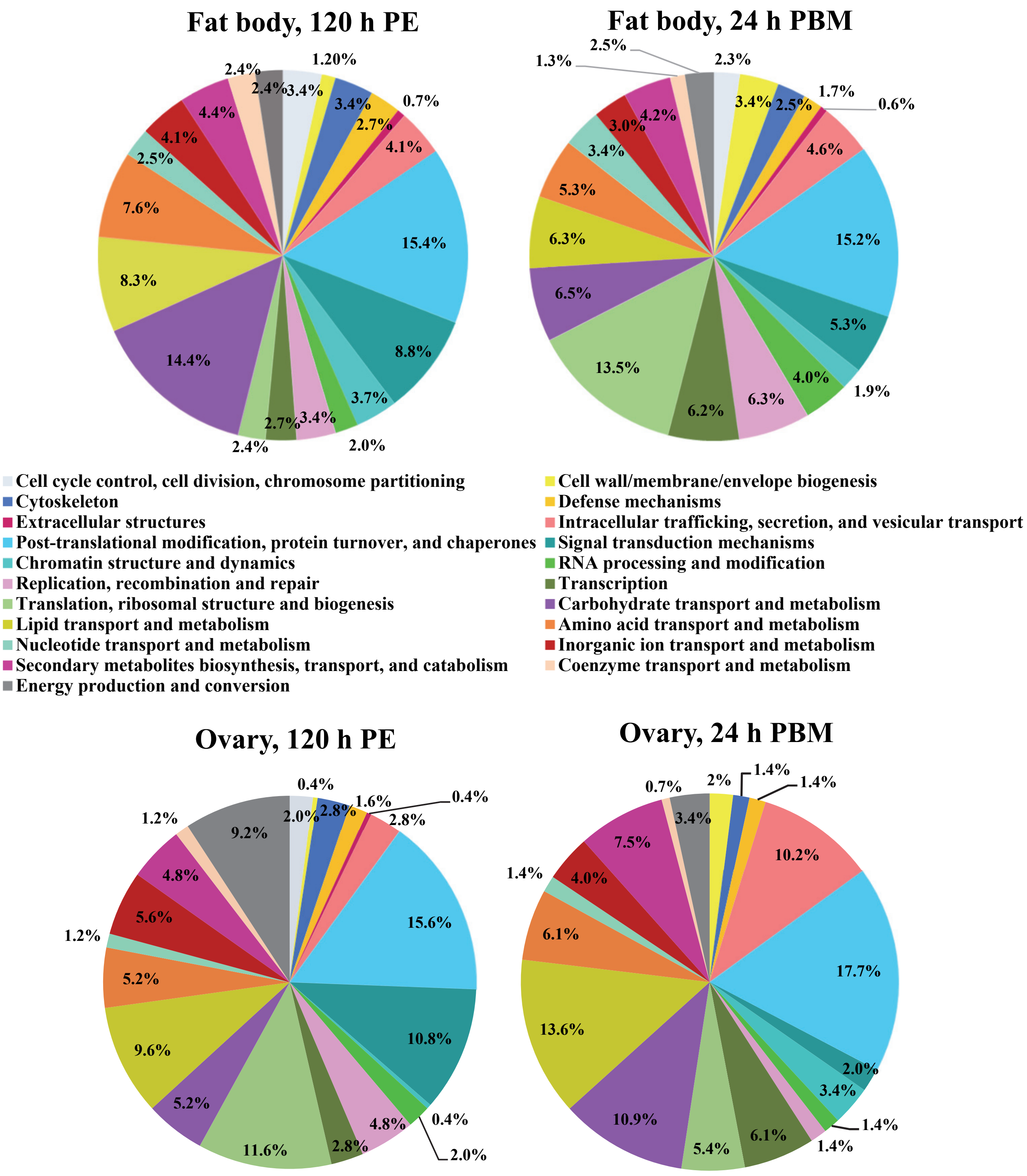

S6 Fig. Differential gene expression in the fat body and ovary after PPF treatment. Adult female mosquitoes were treated with PPF (70 μg/cm^2) at 72 h PE. Cyclohexane was used as a solvent control. RNA-seq analyses were performed using mosquito fat body and ovary tissues collected at 120 h PE and 24 h PBM. The percentage of differentially expressed genes in discrete functional categories is displayed in the pie chart. PE, Post eclosion; PBM, Post blood-meal.
