# Supplementary material for: Molecular action of pyriproxyfen: Role of the Methoprene-tolerant protein in the pyriproxyfen-induced sterilization of adult female mosquitoes
Source: PLoS Negl Trop Dis. 2020 Aug 31;14(8):e0008669. doi: 10.1371/journal.pntd.0008669 (PMC7485974; doi:10.1371/journal.pntd.0008669)
Supplement: S7 Fig — Adult female mosquitoes were treated with PPF at 72 h PE. The expression of selected 20E- and JH- response genes was analyzed using real-time PCR. Fold changes of mRNA abundance in the fat body or ovary of PPF-treated mosquitoes were determined relative to the cyclohexane-treated control group. Data are presented as mean ± SD from three independent replicates. Statistical analysis was performed using paired t-test (ns, p > 0.05; *, p < 0.05; **, p < 0.01; ***, p < 0.001). (PDF) [file pntd.0008669.s007.pdf]

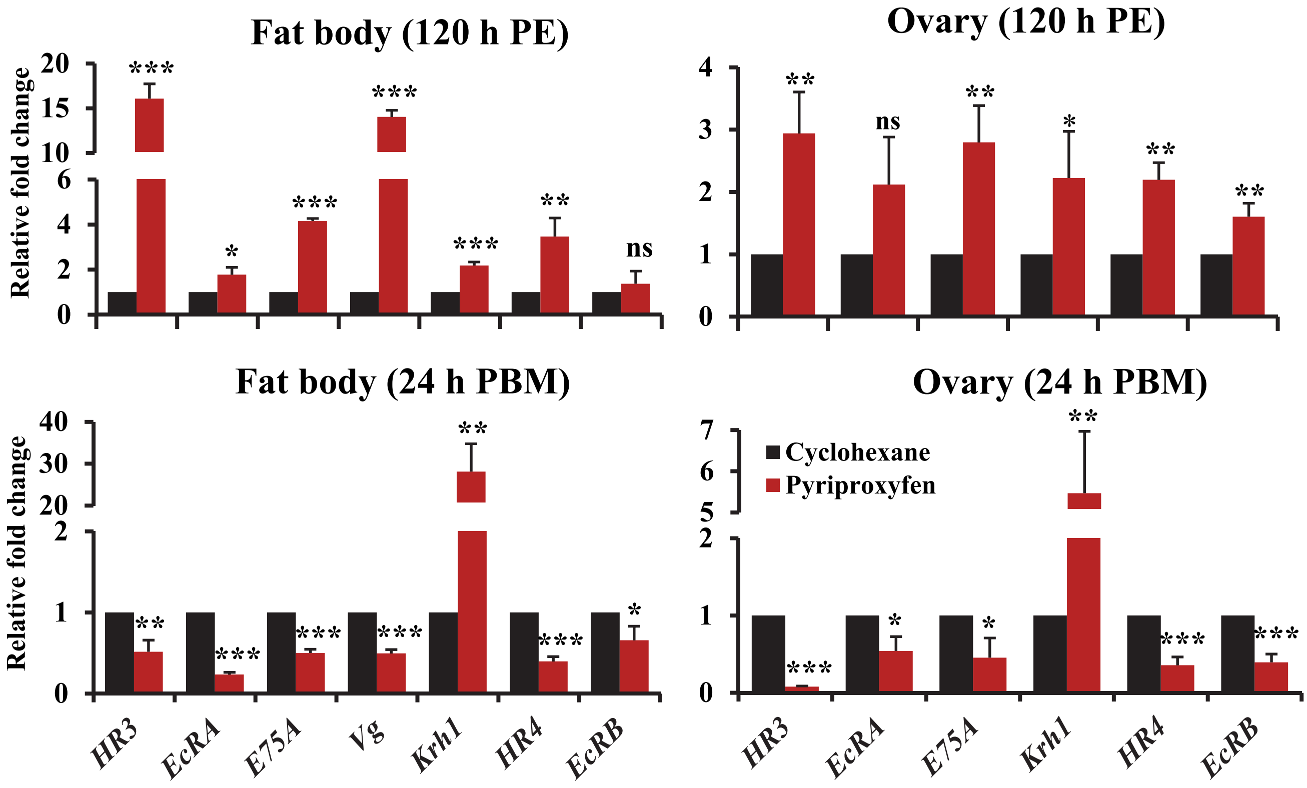

S7 Fig. Expression of 20E response genes in PPF-treated mosquitoes. Adult female mosquitoes were treated with PPF at 72 h PE. The expression of selected 20E- and JH- response genes was analyzed using real-time PCR. Fold changes of mRNA abundance in the fat body or ovary of PPF-treated mosquitoes were determined relative to the cyclohexane-treated control group. Data are presented as mean ± SD from three independent replicates. Statistical analysis was performed using paired *t*-test (ns, $p > 0.05$; *, $p < 0.05$; **, $p < 0.01$; ***, $p < 0.001$).
