# Supplementary material for: Molecular action of pyriproxyfen: Role of the Methoprene-tolerant protein in the pyriproxyfen-induced sterilization of adult female mosquitoes
Source: PLoS Negl Trop Dis. 2020 Aug 31;14(8):e0008669. doi: 10.1371/journal.pntd.0008669 (PMC7485974; doi:10.1371/journal.pntd.0008669)
Supplement: S8 Fig — (A) An overlap between genes that were modulated by PPF treatment and those that were affected by depletion of Met (iMet) in the previtellogenic fat body. The Met-dependent genes were reported by Zou et al. [49]. (B) Scatter plot of the 345 PPF genes that were significantly altered in response to both PPF treatment and RNAi-mediated knockdown of Met. Log2 fold changes (lfc) of iMet (Met RNAi vs control RNAi) were shown in X axis, whereas log2 fold changes after PPF exposure (PPF treatment vs control treatment) were represented in Y axis. Each dot illustrates a single gene. (PDF) [file pntd.0008669.s008.pdf]

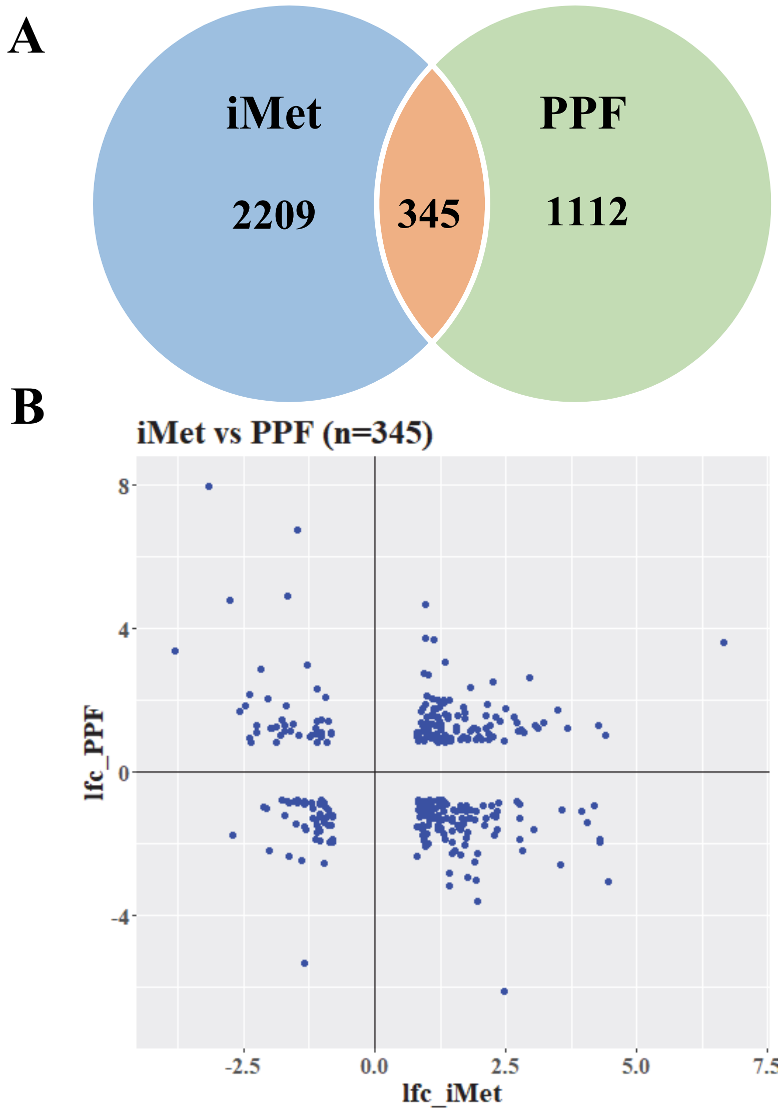

S8 Fig. Differential expression of Met-dependent genes in response to PPF treatment. (A) An overlap between genes that were modulated by PPF treatment and those that were affected by depletion of Met (iMet) in the previtellogenic fat body. The Met-dependent genes were reported by Zou et al. [54]. (B) Scatter plot of the 345 PPF genes that were significantly altered in response to both PPF treatment and RNAi-mediated knockdown of Met. Log2 fold changes (lfc) of iMet (Met RNAi vs control RNAi) were shown in X axis, whereas log2 fold changes after PPF exposure (PPF treatment vs control treatment) were represented in Y axis. Each dot illustrates a single gene.
