# Supplementary material for: Molecular action of pyriproxyfen: Role of the Methoprene-tolerant protein in the pyriproxyfen-induced sterilization of adult female mosquitoes
Source: PLoS Negl Trop Dis. 2020 Aug 31;14(8):e0008669. doi: 10.1371/journal.pntd.0008669 (PMC7485974; doi:10.1371/journal.pntd.0008669)
Supplement: S9 Fig — DsMet was injected into adult female mosquitoes within 1 h after eclosion. Untreated and dsGFP-injected mosquitoes were used as control groups. At 72 h PE, the Met RNAi mosquitoes and control groups were treated with cyclohexane (Cyc) or PPF. Fat bodies were collected at 96 h PE (A) or 24 h PBM (B). Relative mRNA levels of indicated genes were determined using real-time PCR. Error bars represent the standard deviation of three replicates. Statistical significance was conducted using a Student’s t-test (ns, p > 0.05; *, p < 0.05; **, p < 0.01; ***, p < 0.001). (PDF) [file pntd.0008669.s009.pdf]

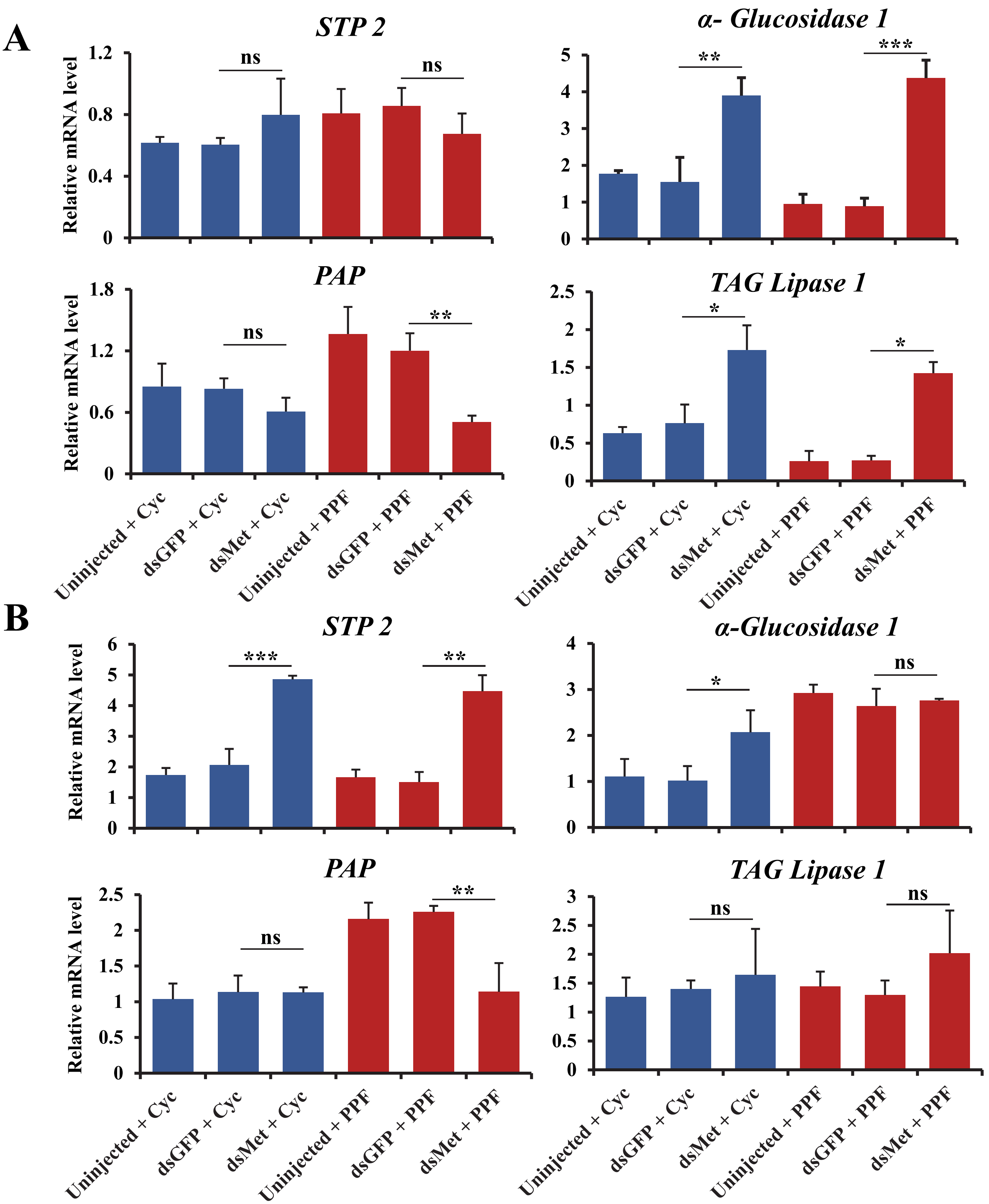

S9 Fig. PPF-triggered alterations of gene expression in the Met-depleted female mosquitoes. DsMet was injected into adult female mosquitoes within 1 h after eclosion. Untreated and dsGFP-injected mosquitoes were used as control groups. At 72 h PE, the *Met* RNAi mosquitoes and control groups were treated with cyclohexane (Cyc) or PPF. Fat bodies were collected at 96 h PE (A) or 24 h PBM (B). Relative mRNA levels of indicated genes were determined using real-time PCR. Error bars represent the standard deviation of three replicates. Statistical significance was conducted using a Student's t-test (ns, $p > 0.05$; *, $p < 0.05$; **, $p < 0.01$; ***, $p < 0.001$).
