# Supplementary material for: Molecular action of pyriproxyfen: Role of the Methoprene-tolerant protein in the pyriproxyfen-induced sterilization of adult female mosquitoes
Source: PLoS Negl Trop Dis. 2020 Aug 31;14(8):e0008669. doi: 10.1371/journal.pntd.0008669 (PMC7485974; doi:10.1371/journal.pntd.0008669)
Supplement: S1 Table — (PDF) [file pntd.0008669.s010.pdf]

S1 Table. Dose-dependent effect of PPF on *Ae. aegypti* reproduction

Groups	% Mortality	% Blood-fed	Eggs/Female	Hatching rate	Relative reproduction rate
**Untreated**	0.0 (±0.0)	98.2 (±1.5)	90.0 (±4.6)	88.0 (±1.0)	-
**Cyclohexane**	0.0 (±0.0)	98.0 (±1.8)	91.6 (±9.5)	84.0 (±1.7)	100
**PPF 3.5 µg/cm^2**	0.9 (±1.6)^{ns}	94.3 (±5.3)^{ns}	90.2 (±5.8)^{ns}	83.0 (±6.2)^{ns}	97.3 (±9.5)^{ns}
**PPF 7 µg/cm^2**	1.0 (±1.7)^{ns}	92.4 (±1.3)*	56.9 (±6.6)**	71.3 (±5.5)*	52.5 (±2.3)***
**PPF 10.5 µg/cm^2**	1.0 (±1.7)^{ns}	91.4 (±2.5)*	56.9 (±9.3)*	33.7 (±3.8)***	24.7 (±2.4)***
**PPF 14 µg/cm^2**	1.1 (±1.9)^{ns}	92.0 (±2.4)*	58.7 (±6.0)**	26.7 (±4.5)***	20.2 (±2.5)***
**PPF 17.5 µg/cm^2**	1.8 (±0.3)^{ns}	92.1 (±1.1)*	48.8 (±8.2)**	24.7 (±5.0)***	16.0 (±5.9)***
**PPF 26.3 µg/cm^2**	1.8 (±1.6)^{ns}	92.5 (±1.6)*	42.0 (±8.2)**	9.9 (±9.5)***	4.8 (±6.0)***
**PPF 35 µg/cm^2**	1.8 (±1.6)^{ns}	92.1 (±2.5)*	20.3 (±10.6)***	5.7 (±2.3)***	1.4 (±0.9)***
**PPF 70 µg/cm^2**	1.8 (±1.6)^{ns}	92.0 (±3.0)*	5.7 (±3.7)***	0.0 (±0.0)***	0.0 (±0.0)***
**PPF 105 µg/cm^2**	7.0 (±3.5)*	92.3 (±1.5)*	7.4 (±7.5)***	0.0 (±0.0)***	0.0 (±0.0)***
**PPF 175 µg/cm^2**	39.1 (±11.4)**	82.0 (±2.7)**	4.7 (±4.0)**	0.0 (±0.0)***	0.0 (±0.0)***

Note: Mortality was measured at 24 h after PPF exposure. After blood-feeding, only fully engorged mosquitoes from each experimental group were kept for the measurements of eggs/female, hatching rate and relative reproduction rate determination. ns, $p > 0.05$; *, $p < 0.05$; **, $p < 0.01$; ***, $p < 0.001$
