# Supplementary material for: Molecular action of pyriproxyfen: Role of the Methoprene-tolerant protein in the pyriproxyfen-induced sterilization of adult female mosquitoes
Source: PLoS Negl Trop Dis. 2020 Aug 31;14(8):e0008669. doi: 10.1371/journal.pntd.0008669 (PMC7485974; doi:10.1371/journal.pntd.0008669)
Supplement: S2 Table — (PDF) [file pntd.0008669.s011.pdf]

S2 Table. Effect of PPF exposure on follicle/egg morphology

Regimens	Treatment	Follicle length (µm)	Egg length (L) (µm)	Egg width (W) (µm)	Egg shape (L/W)
**72 h PE**	**Untreated**	445 (±20.4)	584.9 (±17.9)	171.5 (±13.4)	3.4 (±0.3)
	**Cyclohexane**	456.4 (±25.1)	593.2 (±25.1)	173.7 (±10.1)	3.4 (±0.3)
	**PPF**	211.1 (±13.3)***	446.1 (±42.6)***	183.3 (±10.2)***	2.4 (±0.3)***
**96 h PE**	**Untreated**	445 (±20.4)	584.9 (±17.9)	171.5 (±13.4)	3.4 (±0.3)
	**Cyclohexane**	449.3 (±25.6)	586.9 (±28.7)	169.5 (±8.5)	3.5 (±0.2)
	**PPF**	234.7 (±14.5)***	457.7 (±29.6)***	173.6 (±14.4)^{ns}	2.8 (±0.3)***
**0.5 h PBM**	**Untreated**	445 (±20.4)	584.9 (±17.9)	171.5 (±13.4)	3.4 (±0.3)
	**Cyclohexane**	447.5 (±25.0)	593.7 (±23.6)	171.4 (±11.4)	3.5 (±0.2)
	**PPF**	237.8 (±15.9)***	452.5 (±36.5)***	167.0 (±13.0)^{ns}	2.6 (±0.4)***
**24 h PBM**	**Untreated**	445 (±20.4)	584.9 (±17.9)	171.5 (±13.4)	3.4 (±0.3)
	**Cyclohexane**	449.7 (±23.4)	583.5 (±28.6)	171.5 (±9.5)	3.4 (±0.2)
	**PPF**	351.3 (±31.2)***	535.5 (±33.9)***	169.2 (±10.6)^{ns}	3.2 (±0.3)***
**36 h PBM**	**Untreated**	445 (±20.4)	584.9 (±17.9)	171.5 (±13.4)	3.4 (±0.3)
	**Cyclohexane**	452.1 (±23.1)	595.7 (±19.8)	168.8 (±5.9)	3.5 (±0.1)
	**PPF**	430.4 (±48.7)*	541.0 (±37.6)***	172.2 (±8.2)^{ns}	3.1 (±0.2)***

Note: Statistical differences between the PPF-treated and cyclohexane-treated mosquitoes were analyzed using paired t-test. ns, $p > 0.05$; *, $p < 0.05$; **, $p < 0.01$; ***, $p < 0.001$
