# Supplementary material for: Molecular action of pyriproxyfen: Role of the Methoprene-tolerant protein in the pyriproxyfen-induced sterilization of adult female mosquitoes
Source: PLoS Negl Trop Dis. 2020 Aug 31;14(8):e0008669. doi: 10.1371/journal.pntd.0008669 (PMC7485974; doi:10.1371/journal.pntd.0008669)
Supplement: S3 Table — (PDF) [file pntd.0008669.s012.pdf]

S3 Table. Amounts of circulating sugars after PPF exposure

Groups	Trehalose (µg/ female)	Sucrose (µg/ female)	Glucose (µg/ female)	Fructose (µg/ female)
**120 h PE**				
**Untreated**	5.41 (±0.60)	21.24 (±0.03)	11.36 (±0.44)	5.16 (±0.27)
**Cyclohexane**	5.01 (±0.45)	21.37 (±0.27)	11.22 (±0.37)	5.48 (±0.16)
**PPF**	4.09 (±0.81) ns	23.17 (±0.29)*	13.07 (±0.36)*	7.54 (±0.12)**
**24 h PBM**				
**Untreated**	3.61 (±0.21)	16.07 (±0.81)	6.0 (±0.31)	5.50 (±0.10)
**Cyclohexane**	3.51 (±0.16)	16.11 (±0.57)	6.06 (±0.35)	5.72 (±0.07)
**PPF**	3.50 (±0.08) ns	20.96 (±0.33)**	7.44 (±0.77) ns	6.73 (±1.06) ns

Note: ns, $p > 0.05$; *, $p < 0.05$; **, $p < 0.01$
